# Assessment of Habitat Representation across a Network of Marine Protected Areas with Implications for the Spatial Design of Monitoring

**DOI:** 10.1371/journal.pone.0116200

**Published:** 2015-03-11

**Authors:** Mary Young, Mark Carr

**Affiliations:** 1 Ecology and Evolutionary Biology, University of California Santa Cruz, Santa Cruz, California, United States of America; 2 Centre for Integrative Ecology, Deakin University, Warrnambool, Victoria, Australia; Università di Genova, ITALY

## Abstract

Networks of marine protected areas (MPAs) are being adopted globally to protect ecosystems and supplement fisheries management. The state of California recently implemented a coast-wide network of MPAs, a statewide seafloor mapping program, and ecological characterizations of species and ecosystems targeted for protection by the network. The main goals of this study were to use these data to evaluate how well seafloor features, as proxies for habitats, are represented and replicated across an MPA network and how well ecological surveys representatively sampled fish habitats inside MPAs and adjacent reference sites. Seafloor data were classified into broad substrate categories (rock and sediment) and finer scale geomorphic classifications standard to marine classification schemes using surface analyses (slope, ruggedness, etc.) done on the digital elevation model derived from multibeam bathymetry data. These classifications were then used to evaluate the representation and replication of seafloor structure within the MPAs and across the ecological surveys. Both the broad substrate categories and the finer scale geomorphic features were proportionately represented for many of the classes with deviations of 1-6% and 0-7%, respectively. Within MPAs, however, representation of seafloor features differed markedly from original estimates, with differences ranging up to 28%. Seafloor structure in the biological monitoring design had mismatches between sampling in the MPAs and their corresponding reference sites and some seafloor structure classes were missed entirely. The geomorphic variables derived from multibeam bathymetry data for these analyses are known determinants of the distribution and abundance of marine species and for coastal marine biodiversity. Thus, analyses like those performed in this study can be a valuable initial method of evaluating and predicting the conservation value of MPAs across a regional network.

## Introduction

Human impacts on the oceans continue to increase [1, 2, 3, 4] and several governments throughout the world have acknowledged the need for more ecosystem-based conservation measures in the marine environment [5, 6, 7, 8]. Among these approaches, the use of marine protected areas (MPAs) is becoming widely adopted to protect ecosystems, their biodiversity and to supplement traditional fisheries management [9, 10, 11, 12, 13, 14, 15, 16]. MPAs are areas within the ocean that are spatially protected from differing levels of human impacts, including resource exploitation and habitat alterations. MPAs can conserve habitats and unexploited species in addition to species targeted by fisheries [17, 18, 19, 20].

One major consideration when designing a network of MPAs for the purpose of conserving biodiversity and ecosystems is the representation of habitat and the ability to capture the diversity and heterogeneity of habitat features that support biodiversity [21, 22, 23, 24, 25, 16, 26]. MPAs will only be successful tools for biodiversity conservation if they protect the diversity of habitats that support the variety of ecosystems that generate and sustain the biodiversity targeted for protection. In addition, especially when designing networks of MPAs, replication of habitats among MPAs is required for reducing the likelihood of losing an ecosystem targeted for protection to a natural (e.g., hurricane) or anthropogenic (e.g., oil spill) perturbation, contributing to larval connectivity of species populations and communities across the network, and for the analysis and evaluation of MPA effects to inform their adaptive management [18, 11, 26, 27, 28]. Also, for MPAs to contribute to a network based on larval connectivity, individual MPAs have to contain enough habitat to support large enough populations to provide sufficient larval production, and MPAs have to be spaced at appropriate distances to one another. Therefore, representation and replication of sufficiently sized habitats across MPA networks has major implications for the ecological connectivity of populations and the resulting effectiveness of the network [26, 27, 28].

Seabed mapping, both *in situ* and remotely sensed, has emerged as a much needed tool to determine the level of representation of the different habitat types inside and outside MPAs [23, 29, 30]. The application of seafloor maps to characterize habitat is based on established species, community or ecosystem associations with combined geomorphological (e.g., substratum type, relief, rugosity) and oceanographic features (e.g., water depth, currents, wave exposure). Habitat assessments using *in situ* observations such as SCUBA or remotely operated vehicles (ROVs) are widely utilized but are limited in their depth ranges and the ability to efficiently sample large areas [31]. With the advent and improvements in remote sensing equipment and techniques, remote sensing in the marine environment has become a very efficient and cost effective means for comprehensive mapping by covering large areas of the ocean floor at high resolution [32, 33, 29, 34]. Once mapped, the seafloor can be combined with biological data and characterized into distinct habitat classes to be used for the placement of new and assessment of already existing MPAs at the scale of entire networks.

Products produced from remotely-sensed data have become fundamental to many applications of coastal marine science [30]. Maps generated from seafloor mapping have been used to help identify essential habitat for many commercially important species [35, 36, 30] and to identify habitats associated with biodiversity “hotspots” [30]. Cogan et al. [29] states that marine habitat mapping should be the “launch point” for ecosystem-based management by allowing for the characterization of habitat features across the ecosystem of interest. Application of marine habitat maps for generating new information on marine ecosystems and for the design of new or evaluation of existing MPAs is increasing [37, 38, 39]. These habitat maps help to spatially integrate information including remotely sensed data, biological observations, human use, vulnerability, environmental quality, etcetera [40, 39]. However, when biological data do not exist to create detailed habitat maps, surrogates, including geomorphological information, can be used to provide information on potential habitats [41, 42]. For example, Rees et al. [43] assessed the ability to use remotely-sensed bathymetric data as surrogates for temperate reef communities and found that, although the seafloor structure did not explain variation in reef fishes, the abiotic surrogates explained much of the variation in the invertebrate communities. Although it is preferable to have all the data necessary to develop detailed habitat maps over large areas of the seafloor, acquiring all types of data over broad scales in the oceans is difficult. Therefore, the use of abiotic surrogates for characterizing marine ecosystems and for marine protected area planning has the potential to increase the effectiveness of MPAs.

The state waters of California offer a unique opportunity to develop methods to assess the representation of habitat or habitat surrogates inside and outside of MPAs across a regional network and the replication of those habitats. Not only has California adopted the Marine Life Protection Act (MLPA), which produced a statewide network of MPAs that extends along the 1200 km of coastline from the Oregon border to the Mexico border and out to the three nautical mile limit of state waters [44], they also implemented the California State Mapping Program (CSMP). The CSMP is a statewide mapping program resulting in a high-resolution geologic basemap for much of the 14,500 square kilometers of California state waters [45]. The high-resolution data from the CSMP consist of digital elevation models (DEMs) from sonar mapping of the seafloor. These DEMs provide depth information as well as information on the structure of the seafloor. For this study, we used the multibeam echosounder (MBES) and inter-ferometric sonar data because they provided the best available seafloor data for the region of interest.

The Central Coast Region of California is a particularly valuable example for evaluating the representation of habitats across the network of MPAs created by the MLPA design process. The Central Coast Region was the first section of the statewide network to be designed and preceded the high resolution seafloor mapping conducted by the CSMP. During the design of the Central Coast Region, seafloor information was incomplete and in the absence of comprehensive seafloor mapping, a variety of proxies were used including aerial images of the surface canopy of kelp forests as proxy for nearshore rock, and rockfish “fishing areas” based on recreational and commercial fishing landings as a proxy for offshore rocky reefs. Analysis of seafloor maps eventually produced in the central coast provides an opportunity to evaluate how well those proxies accurately represented the distribution and types of seafloor features, both across the region and within the MPAs created in that region.

One hallmark of the structure of the MLPA design process was the role of stakeholders. These were individuals with vested interests in outcome of the network design, including recreational and commercial fishermen, conservation groups, state and federal management agencies and many others (http://www.dfg.ca.gov/marine/mpa/centralcoast_rsg.asp). Stakeholders designed the network of MPAs based on ecological guidelines (e.g., size, spacing, habitat representation) provided them by a science advisory team, but were also allowed to consider socio-economic consequences of their design. As such, in the Central Coast and other regions of the statewide network, the design of individual MPAs and the network deviated from that prescribed by the science guidelines. Although habitats were identified for representation (see Methods section), stakeholders were not given a target proportion of any habitat to include in the network. Moreover, the amount of area of each habitat considered adequate to constitute a replicate of that habitat within an MPA was not determined until after the Central Coast design process was completed. Nonetheless, to determine how well the Central Coast design might achieve its conservation goals, it is instructive to determine how well that process met the guidelines that were generated for and applied to subsequent regions of the statewide network.

There is also a need to evaluate the design of monitoring programs that are being used to assess the efficacy of networks of MPAs. Many fish populations have been shown to vary in abundance based on the three-dimensional structure of their environment [46, 47, 48, 49]. It is important, therefore, for MPA monitoring programs to characterize and account for variability in that structure when comparing biological data collected inside and outside MPAs [50, 47, 51] so that differences in populations and ecosystems can be attributed to the effect of the MPAs rather than confounded by differences in habitat characteristics [47]. The Partnership for Interdisciplinary Studies of Coastal Oceans (PISCO) has characterized fish populations and kelp forest ecosystems within the nearshore (0–20m depth range) of the Central Coast MLPA region. The basic monitoring design for this region includes kelp forest sites within and outside each MPA. These monitoring sites, however, were set up with little knowledge of the underlying seafloor structure because the seafloor data did not exist at the time the monitoring sites were chosen.

The purpose of this study is to use multibeam bathymetry data acquired by the CSMP and the baseline monitoring data acquired by PISCO to evaluate the distributions of habitat defined in the MLPA process in the Central Coast MPA network and the representation of reef structure in the kelp forest monitoring design. To evaluate the placement of MPAs within the Central Coast network, we classified the seafloor data from the CSMP into potential habitat variables of known importance to many species (i.e., depth, rugosity, slope, etc.) and used this information on seafloor structure to answer the following questions for the region:

1. How well does the current network of MPAs representatively (i.e. proportionately) capture the habitat types designated for protection by the MLPA design process?
2. How well are MLPA habitats replicated throughout the region (i.e. is there sufficient habitat within MPAs to contribute to population connectivity)?
3. How well are classes of geomorphological features based on finer scale topographic metrics, which were not considered when the MPAs were designed, represented across the MPA network?
4. Does the kelp forest monitoring program representatively sample the diversity of seafloor structure inside the MPAs?
5. How well does seafloor structure within reference sites outside MPAs match those sampled within the MPAs?

The null hypotheses are that relative amounts of habitat within the MPAs are representative of (proportional to) the region, habitats are adequately replicated with sufficient habitat area based on the guidelines in the MLPA, and that the kelp forest monitoring program has adequately captured all available habitats in their monitoring design. Therefore, we test for deviation from these hypotheses.

## Methods

**Fig 1:**
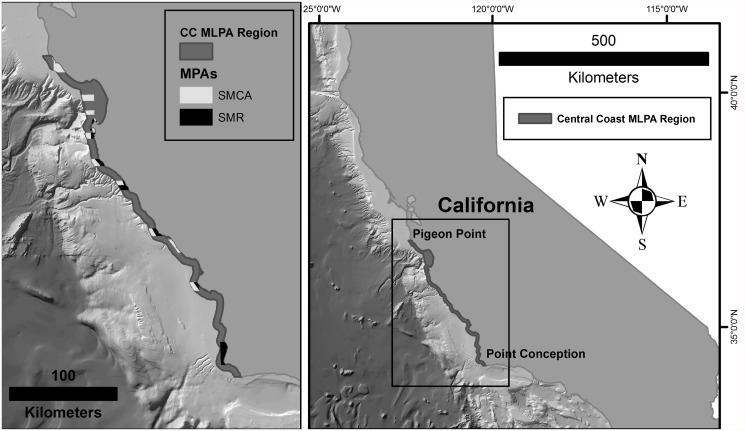
Central Coast MLPA Region. Image on the left is the Central Coast MLPA region along the Central Coast of California and the MPAs within the region. SMCA are State Marine Conservation Areas with limited allowable take, SMR are State Marine Reserves with no recreational or commercial take. The Central Coast MLPA Region extends three nautical miles (boundary of state waters) from shore. The image on the right shows where this region falls along the California coast.

The study site for this project is along the central coast of California in the Central Coast MLPA region (http://www.dfg.ca.gov/marine/mpa/ccmpas_list.asp). This region extends from Pigeon Point in the north (37°10′57“ N 122°23′38” W) to Point Conception in the south (34°26′55” N 120°28′14” W) and consists of a network of 29 MPAs with differing levels of protection (Fig. 1).

### MLPA Habitat Classification

**Fig 2:**
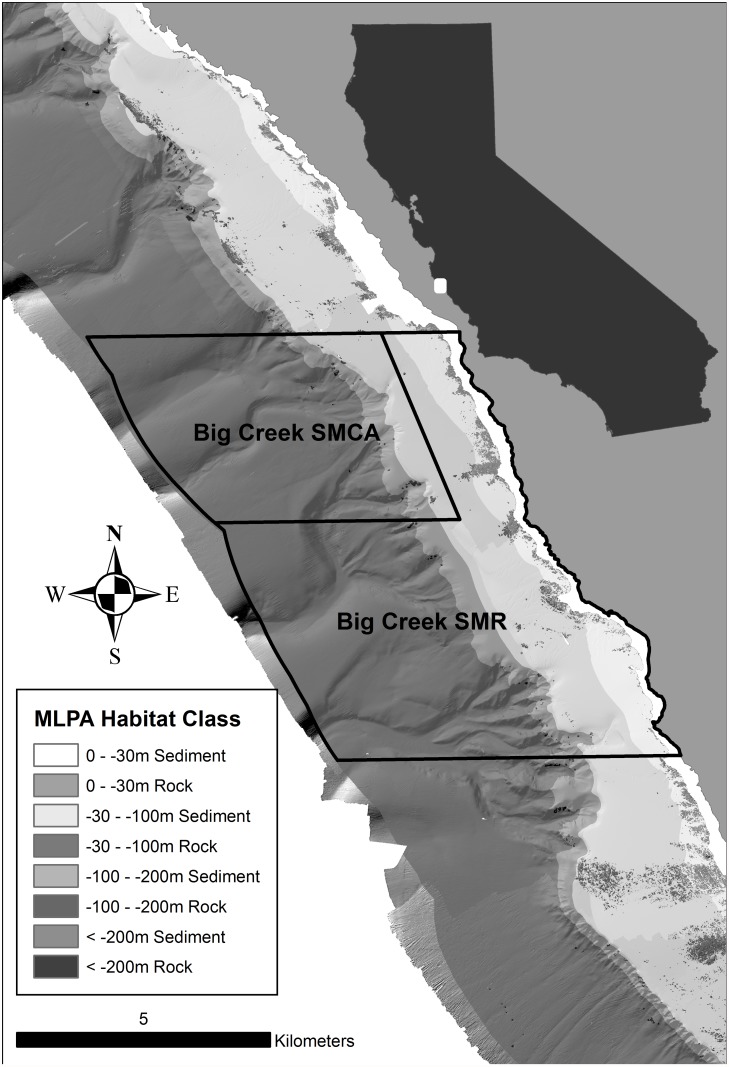
Example of results from the seafloor habitat classification within and around the Big Creek MPA. The different shades of gray represent the different substrate types and depth zones.

In the MLPA master plan, eight habitat classes were developed for the subtidal that described the substrate and depth zones that are associated with specific distributions of species and communities [52, 53, 26]. These habitat classes were based on two substrate types (soft sediment vs. rock) in four depth zones (0–30m, 30–100m, 100–200m and greater than 200m). These depth zones and substrates are often associated with changes in species composition and were used as the delineators for the MLPA habitat classes (e.g., Fig. 2).

The MLPA habitat products were created using the DEMs from the multibeam and inter-ferometric bathymetry data. These data are publicly available and can be downloaded from the CSMP website: http://seafloor.otterlabs.org/csmp/csmp.html. A DEM is a raster dataset with elevation values at regularly spaced intervals. The DEMs varied in resolution based on the range of bottom depths they captured (0–85m at 2m resolution, 85–250m at 5m resolution, and >250m at 10m resolution). Each of the cells in these DEMs were classified into the MLPA habitat categories by first assigning “sediment” or “rock” substratum types based on thresholds of ‘ruggedness’ and then breaking them up by specified depth ranges (Appendix A in S1 File). The resulting classified maps were resampled to 5m for consistency in resolution for further analyses.

### How well does the current network of MPAs representatively (i.e. proportionately) capture the habitat types designated for protection by the MLPA design process?

**Fig 3:**
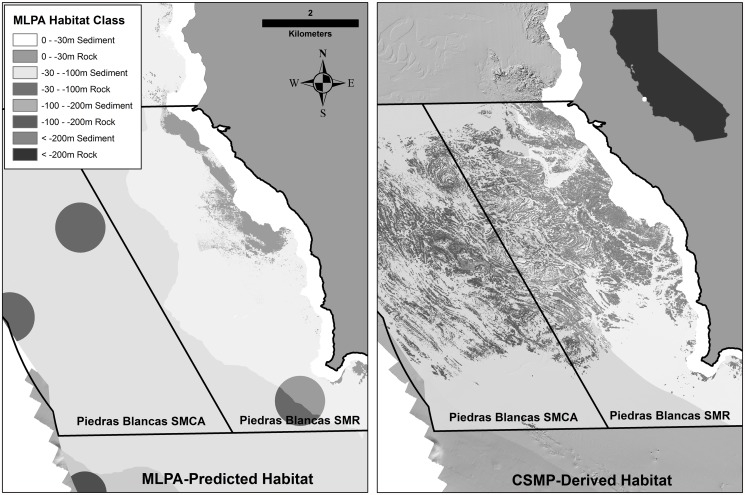
Comparison of the MLPA-predicted habitat classifications using the best available data during the designation of the MPAs (left) and CSMP-derived habitats (right) in the Piedras Blancas MPA. The MLPA-predicted habitats were derived from proxies for rock such as kelp forest coverage, rockfish fishing areas (the circular features in the image), or broad-scale predicted substrate maps. The CSMP-derived habitats were created based on the rugosity of the surface as a proxy for rock or sediment using the high-resolution digital elevation models from the CSMP data.

Using MLPA habitat class maps, we quantified the proportionate availability of each habitat category across the entire region and the proportionate representation within the MPAs. First, we created an ESRI shapefile defining the extent of the Central Coast Region. Then, using the *Tabulate Area* tool within the *Spatial Analyst* toolbox in ArcGIS, we quantified the total area of each habitat class within the region. To quantify the habitat within the MPAs, we used the shapefile of the MPA boundaries provided by the California Department of Fish and Wildlife and, using the same methods as for the region, we tabulated the area of each habitat class within the MPAs. To test the hypothesis that each habitat was adequately represented across the network of MPAs, we compared the proportion of each habitat category captured across the network with the proportion of that habitat available across the entire region. A common threshold when comparing percentages is 20% [54]. Therefore, we rejected the hypothesis that the current network of MPAs representatively (i.e. proportionately) capture the habitat types designated for protection by the MLPA design process if the percentage of any habitat across the network deviated from the percentage of that habitat category across the study region by more than 20%. In addition, to determine how well representation of each habitat category was estimated during the MLPA process (i.e. before availability of the seafloor maps), we compared our estimates of proportionate representation based on the seafloor maps with estimates of representation generated from the MLPA- predicted habitat available at the time that the MPAs were designed. These MLPA-predicted habitat maps were based on proxies for rock, sediment, and depth using the best available data at the time. For example, kelp coverage was used as a proxy for shallow rocky habitat, while the locations where fishermen had caught rockfish were used as proxies for rocky habitat in the deeper water. Fig. 3 provides a graphical representation of the comparison of the MLPA-predicted habitat and the CSMP-derived habitat within a single MPA. The nearshore area in the MLPA-predicted habitat map shows areas of predicted rocky reef where kelp occurs along with the circular “rock” areas represented as a buffered area around common rockfish fishing areas. The CSMP-derived habitat shows areas delineated as rock and sediment by depth based on features in the high resolution multibeam data (Fig. 3) [53].

### How well are the MLPA habitats replicated throughout the region?

Deviations from proportional representation of habitat types in MPAs is particularly problematic if there is insufficient representation of a habitat within MPAs to achieve replication (i.e. sufficient habitat area to support populations that will contribute to larval production and connectivity of populations associated with that habitat). If individual MPAs are found to contain an inadequate area of habitat, they may not contribute to the network [53]. Guidelines for the minimum abundance of a habitat within an MPA to qualify for replication of that habitat were generated by the MLPA Science Advisory Team for the North Central Coast Region only after completion of the Central Coast Region [55]. However, these guidelines were calculated from species-area curves based on ecological surveys conducted in the Central Coast Region and were developed for only four of the eight MLPA-habitat classes. The minimum area required to include 90% of the species in each habitat was the basis for the guideline. For shallow (0–30m depth) habitats (e.g., rocky reef, kelp forests and sand bottom), the guideline was a linear distance of habitat along the coast (1.8 km). Species-area curves were available for kelp forests, not shallow rocky reef, so the same guideline was applied to both habitats. Deeper (30–100m depth) habitats were area-based; deep rocky reef and sandy bottom were 0.52 km^2^ and 26 km^2^, respectively. To assess the adequacy of habitat within each MPA, we used the CSMP-derived habitat maps to measure the linear distance of shallow (0–30m depth) rocky reef and sandy bottom habitat, and the area (km^2^) of deeper (30–100m depth) rocky reef and sandy bottom. Replication guidelines were only available for the 0–30m and 30–100m habitats so only four MLPA habitats within those depth zones were assessed for replication. The linear distance measurements for the kelp forest habitat were derived from maps of the maximum coverage of kelp using LANDSAT data [56, 57]. Linear distance of shallow habitats was measured along the 15m depth isobath. In addition, the MLPA specifies that there needs to be a minimum of three, ideally five, replicate MPAs for each of the habitat classes [53]. Thus, we rejected the hypothesis that the current network of MPAs had sufficient replication of each habitat category if our estimates of habitat area in each MPA identified fewer than three MPAs with sufficient habitat area to meet the area guidelines required for that habitat [53].

### How well are regional geomorphic classes based on finer scale topographic metrics, which were not considered when the MPAs were designed, represented across the MPA network?

As specified in the MLPA, substrate (i.e. rock or sediment) was deemed an important habitat factor affecting the distribution of species. However, habitat structure, including habitat complexity and heterogeneity, has also been shown to affect variation in fish population size and assemblage structure [58, 59, 60, 61]. There are clear distinctions in rocky reef structure along the coast of California that are evident in the CSMP data (Fig. 3). It is important to consider these finer scale differences in seafloor structure when assessing representation of habitat within MPAs. Accordingly, we classified rocky reefs along the Central Coast Region based on slope, rugosity, and topographic position index (TPI) classes (Appendix B in S1 File). These reef characteristics have been shown to explain some of the variation in species-habitat associations across rocky reefs [61, 36].

Once we classified rocky reef into distinct geomorphic classes, we tabulated the area of each of those classes for both the regions and for MPAs within the region to determine representation of those geomorphic classes across MPA networks. We then compared the proportionality of each of these fine-scale geomorphic classes across the region to the proportions within the MPAs to see how well these classes represented their availability throughout the region. Again, we used a 20% difference threshold to determine if there were significant differences between the percentages of each of the habitat classes found throughout the region compared to the percentages observed in the MPAs [54]. We rejected the hypothesis that the current network of MPAs representatively (i.e. proportionately) capture the fine-scale geomorphic classes if the percentage of any habitat across the network deviated from the percentage of that geomorphic class across the study region by more than 20% [54].

### Does the kelp forest monitoring program adequately capture the variability in seafloor structure inside the MPAs and does the monitoring program sample the same seafloor structure inside and outside MPAs?

Because species assemblages vary across habitat types, it is important to design MPA monitoring programs to incorporate representative habitat inside and outside of MPAs so that any differences are not confounded by the sampling of different proportions of habitat types. We assessed habitat representation in the kelp forest monitoring dataset, which is only one of the studies used for baseline characterization of the Central Coast MLPA region. The kelp forest monitoring program (PISCO) relies on divers and, therefore, samples habitats in depths shallower than 20 meters. This analysis provides an example of how seafloor data can be used to help stratify monitoring locations across variations in rocky reef.

To determine the habitat representation in the kelp forest monitoring design, we used GPS waypoints along with the initial diver recorded compass heading for the kelp forest survey transects to estimate the location where the transects were conducted in ArcGIS. To do this, we created a 100m polyline from the waypoint utilizing the initial heading of the diver and adjusted to the bathymetric contours, as specified in the PISCO sampling protocols <http://www.piscoweb.org/research/science-by-discipline/ecosystem-monitoring/kelp-forest-monitoring/subtidal-sampling-protoco>. We then placed a 10m buffer around each transect to incorporate the spatial uncertainty in the exact location of that transect, with the assumption that each transect was conducted within that 10m buffer. In addition to the PISCO transects, we created 3,250 transects with the same dimensions as the diver transects that we placed randomly throughout the region in rocky reefs at comparable depths of the kelp forest surveys. These transects allowed us to characterize variation in rocky reef across the region and within MPAs to determine the availability of fine-scale features, which could then be used to assess how well the kelp forest monitoring surveys representatively sampled these features.

Once we created the buffered survey and random transects, we quantified the structure of the rocky reef within each transect using the same fine-scale geomorphic categories that were used for the geomorphic classification. After quantifying the reef structure within each of those transects, we used a cluster analysis within the statistical software package Primer to designate separate classes. Once clustered, we searched the dendrogram for the appropriate merging distance to categorize transects into a number of reef structure classes. Although the MLPA did not take into consideration this scale of variation across rocky reef, these reef structure classes were defined by certain combined characteristics of the rocky reef that were previously shown to be important to the distributions of fish [61, 36, 49]. Incorporating this variation in reef structure into the monitoring design could help to stratify the monitoring transects and sample greater species diversity. The numbers of random and PISCO transects that fell in each reef structure class from the cluster analysis were used to characterize the percentage of each class across the region and within the MPAs. The comparison between the percentage availability of each reef structure class and the percentage of monitoring transects that fell in each class was used to assess how well the kelp forest monitoring program proportionately captured the available classes within the MPAs. In addition, the reef structure classes sampled outside MPAs in their associated reference sites were compared to the reef structure classes sampled in the corresponding MPAs to determine if similar proportions of each reef structure class was sampled in both areas. Again, we rejected the hypothesis that the current monitoring design representatively samples the available reef structure classes within the MPAs and that the reference sites sample the same proportion of reef classes as in the MPAs if they deviated from each other by more than 20% [54].

## Results

### How well does the current network of MPAs representatively (i.e. proportionately) capture the habitat types designated for protection by the MLPA design process?

Across the Central Coast Region, abundance of the four MLPA-designated habitat categories in the 0–100m depth range (based on substrate classification derived from the CSMP data) were similar to the MLPA-predicted habitat from the best available data during the MLPA design process (Table 1). None of these differences between MLPA habitat areas observed from the seafloor maps and those predicted during the design process exceeded the 20% threshold. However, where comparisons could be made at deeper depths (>100m), differences in MLPA habitat abundance estimated from the CSMP data and the MLPA-predicted habitat did exceed the 20% threshold of dissimilarity and there was mismatch in one habitat (100–200m rock), which existed in the MLPA-predicted habitat but was not observed in the CSMP data (Table 1). The absolute differences in CSMP-derived habitat and MLPA-predicted habitat areas ranged from 0–4%. Sediment was the most dominant substrate type in all depth zones making up a total of 91% of the mapped state waters of the Central Coast Region, with the largest percentage falling in the 30–100m depth zone. Rocky habitat, compared to sediment, makes up a much smaller percentage of the mapped state waters (9%) with the largest percent cover falling in the shallowest depth range (0–30m). Across the Central Coast Region, shallow (0–30m depth) sediment and rock were over- and under- predicted in the MLPA-predicted habitat by 3% and 1%, respectively (Table 1). This level of difference (≤ 3%) was similar for both sediment and rock across the deeper depth zones as well. Within the MPAs, there was no difference in the CSMP-derived habitat and the MLPA-predicted habitat in areas of shallow (0–30m depth) sand and rock, and only 1% difference in sand or rock in the 30–100m depth zone (Table 1). With the exception of sediment at depths greater than 200m, all other deeper (> 100m) habitat categories differed by no more than 2% cover (Table 1).

**Table 1:** Percentage of each habitat type across the central coast region and within the Central Coast MPAs predicted by the MLPA Science Advisory Team (SAT) in the design process and the values derived from the CSMP seafloor habitat classification, along with percent deviations between those values for the region and the MPAs.

	Central Coast Region			Central Coast MPAs		
Habitat	MLPA Predicted	CSMP Derived	Percent Deviation	MLPA Predicted	CSMP derived	Percent Deviation
0–30m Sediment	24%	21%	-12.5%	20%	20%	0%
0–30m Rock	6%	5%	-16.7%	8%	8%	0%
30–100m Sediment	49%	51%	4.1%	43%	42%	-2.3%
30–100m Rock	4%	4%	0%	6%	5%	-16.7%
100–200m Sediment	5%	7%	**40%**	7%	9%	**28.6%**
100–200m Rock	1%	0%	**n/a**	3%	0%	**n/a**
>200m Sediment	9%	12%	**33.3%**	11%	15%	**36.4%**
>200m Rock	1%	0%	**n/a**	2%	0%	**n/a**

The bold text identifies those habitat categories whose differences exceeded the 20% deviation threshold and also those habitat categories predicted to exist in the design process but was not detected in the CSMP seafloor data.

**Fig 4:**
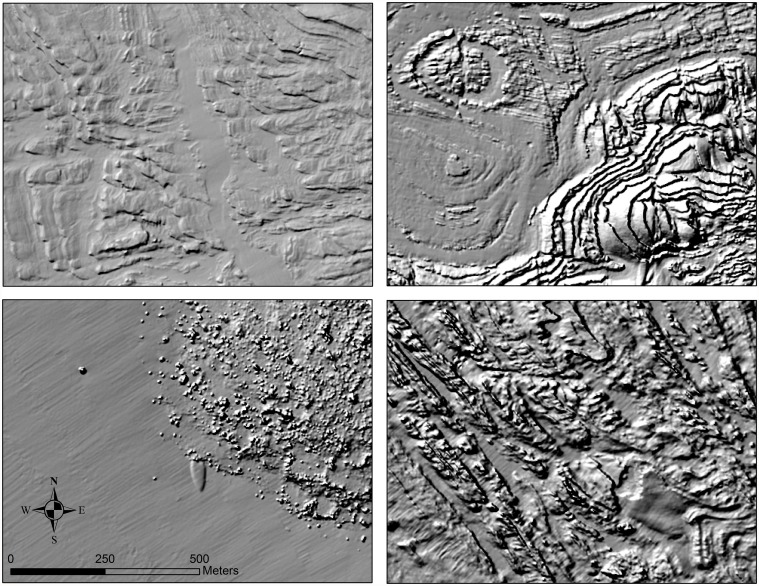
Shaded relief imagery of the seafloor produced from the digital elevation models (2m resolution, Sun Azimuth: 315, Sun Altitude: 45, Z-Factor: 3). These images show the ecologically relevant variation in the structure of rocky reef along the central coast of California.

Although the best data available when setting up the MPAs provided good region-wide estimates of percent cover of habitat in shallow depth zones, the CSMP data revealed that, at the scale of individual MPAs, there were some very large differences in the MLPA-predicted percentage of habitat and the CSMP-derived habitat estimates across all depth zones. The percentage deviation between the MLPA-predicted and CSMP-derived substrate coverage of rock and sediment ranged from 0.2% to 332.8% across the Central Coast MPAs with an average deviation of 39.1% (Table 2). The Piedras Blancas MPA is a good example of where the MLPA predictions grossly under-estimated the area of rocky reef within the MPA and over-estimated soft sediment. In this case, the CSMP data revealed over three times as much rock as what was originally predicted by the MLPA (Table 2; Fig. 4). In fact, at the scale of individual MPAs, there were large differences for many of the MPAs in the amount of MLPA-predicted habitat compared to CSMP-derived habitat (Table 2). Only four of the 23 MPAs used in this analysis fell below the percent deviation threshold of 20% when comparing the MLPA-predicted and CSMP-derived coverage of rocky reef. The MLPA predictions for sediment were slightly better, but only 14 of the 23 MPAs had MLPA-predicted and CSMP-derived coverage within the 20% deviation threshold.

**Table 2:** Percentage of each substrate type within the individual MPAs in the Central Coast region predicted by the MLPA Science Advisory Team (SAT) ("Predicted") compared to the values derived from the CSMP seafloor substrate classification (“Observed”).

MPA	Substrate					
	Rock			Sediment		
	Predicted (%)	Observed (%)	Deviation (%)	Predicted (%)	Observed (%)	Deviation (%)
Año Nuevo SMCA	28.4	39.5	**39.1**	71.6	60.5	-15.5
Greyhound Rock SMCA	10.4	10.6	1.9	89.6	89.4	-0.2
Soquel Canyon SMCA	22.4	1.4	**-93.8**	77.6	98.6	**27.1**
Portuguese Ledge SMCA	32.0	5.1	**-84.1**	68.0	94.9	**39.6**
PGMG SMCA	74.3	55.1	**-25.8**	25.7	44.9	**74.7**
Asilomar SMR	71.5	59.9	-16.2	28.5	40.1	**40.7**
Lover’s Point SMR	35.9	26.9	**-25.1**	64.1	73.1	14.0
Edward F. Ricketts SMCA	31.5	17.9	**-43.2**	68.5	82.1	19.9
Carmel Pinnacles SMR	83.4	75.8	-9.1	16.6	24.2	**45.8**
Carmel Bay SMCA	43.0	33.2	**-22.8**	57.0	66.8	17.2
Point Lobos SMCA	33.7	5.6	**-83.4**	66.3	94.4	**42.4**
Point Lobos SMR	39.2	44.1	12.5	60.8	55.9	-8.1
Point Sur SMR	55.3	36.2	**-34.5**	44.7	63.8	**42.7**
Point Sur SMCA	18.6	11.0	**-40.9**	81.4	89.0	9.3
Big Creek SMCA	1.2	0.3	**-75.0**	98.8	99.7	0.9
Big Creek SMR	4.9	2.5	**-49.0**	95.1	97.5	2.5
Piedras Blancas SMR	12.5	33.6	**168.8**	87.5	66.4	**-24.1**
Piedras Blancas SMCA	6.4	27.7	**332.8**	93.6	72.3	**-22.8**
Cambria SMCA	22.8	29.5	**29.4**	77.2	70.5	-8.7
White Rock (Cambria) SMCA	38.5	48.4	**25.7**	61.5	51.6	-16.1
Point Buchon SMR	17.2	21.4	**24.4**	82.8	78.6	-5.1
Point Buchon SMCA	6.1	3.2	**-47.5**	93.9	96.8	3.1
Vandenberg SMR	8.1	5.6	**-30.9**	91.9	94.4	2.7

Those deviation values highlighted in bold represent those values that exceed the 20% threshold of dissimilarity.

### How well are the MLPA habitats replicated throughout the region?

The CSMP-derived classifications of the MLPA habitat were also used to determine the number of replicates of four of the habitat classes contained within the Central Coast network of MPAs. Three of the four habitat classes are adequately replicated within the MPAs as specified in the MLPA guidelines (Table 3). Based on the linear distance guidelines for rock, sediment, and kelp in the 0–30m depth range, 13, 8, and 9 MPAs serve as replicates, respectively. In addition, the rock habitat within the 30–100m depth range has sufficient replication with a total of 10 replicates. The only habitat that does not reach the minimum number of three replicates is sediment habitat in the 30–100m depth range with only two MPAs containing enough area of sediment in that depth range to serve as a replicate.

**Table 3:** Linear distances for the habitat types in the shallow (0–30m) depth range (rock, sediment and kelp) and areas of habitat types in the deep (30–100m) depth range (rock and sediment) within each MPA along the Central Coast MLPA region.

	Shallow Habitat Linear Distance (km)			Deep Habitat Area (km^2^)	
MPA	Rock	Sediment	Kelp Habitat	Rock	Sediment
Lovers Point SMR	**0.1**	**1.2**	**0.0**	n/a	n/a
Piedras Blancas SMR	4.6	5.4	3.3	1.390	**5.823**
Piedras Blancas SMCA	n/a	n/a	n/a	6.080	**16.270**
Carmel Pinnacles SMR	n/a	n/a	n/a	0.763	**0.287**
Edward F. Ricketts SMCA	**0.3**	**1.1**	**0.2**	n/a	n/a
Carmel Bay SMCA	2.5	2.2	4.1	**0.322**	**0.922**
Point Lobos SMR	4.1	**1.5**	4.5	3.583	**5.314**
Point Lobos SMCA	n/a	n/a	n/a	0.557	**0.211**
Ano Nuevo SMCA	5.9	3.7	0.0	2.030	**4.241**
PGMG SMCA	2.0	**0.9**	**1.7**	**0.251**	**4.241**
Asilomar SMR	2.3	**0.8**	1.8	**0.156**	**0.061**
Soquel Canyon SMCA	n/a	n/a	n/a	**0.350**	38.143
Portuguese Ledge SMCA	n/a	n/a	n/a	**0.337**	**4.177**
White Rock SMCA	3.5	2.0	5.3	**0.256**	**1.043**
Cambria SMCA	5.2	4.3	7.4	**0.002**	**0.378**
Point Sur SMR	7.0	4.7	8.1	2.471	**7.541**
Point Sur SMCA	n/a	n/a	n/a	2.832	**22.397**
Point Buchon SMR	4.4	**0.7**	3.5	1.229	**11.809**
Point Buchon SMCA	n/a	n/a	n/a	0.837	**20.991**
Greyhound Rock SMCA	4.3	**0.7**	**0.0**	**0.090**	**22.302**
Big Creek SMR	2.6	6.8	5.6	**0.151**	**8.212**
Big Creek SMCA	n/a	n/a	n/a	**0.020**	**2.612**
Vandenberg SMR	3.4	19.6	**0.0**	**0.219**	26.724

Those percentages that do not meet the criteria to be considered a replicate as specified in the MLPA (1.8 kilometers for 0–30m rock, sediment and kelp; 0.52 km^2^ for 30–100m rock; 26 km^2^ for 30–100m sediment) are in bold typeface. “n/a” indicates a depth range is not present in an MPA and, therefore, that habitat is not present in the MPA.

### How well are regional geomorphic classes based on finer scale topographic metrics, which were not considered when the MPAs were designed, represented across the MPA network?

**Fig 5:**
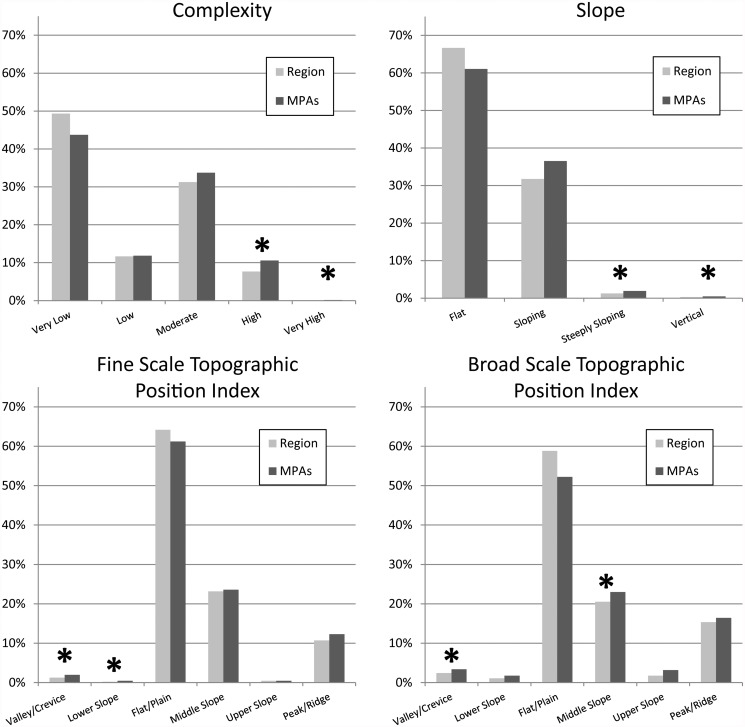
Comparison of the percentage of habitat classes derived from the CSMP data across the region (light gray) and within the MPAs (dark gray). The asterisks (*) above the bars represent those pairs that fell outside the 20% threshold of similarity.

The relative abundance of geomorphic classes based on fine-scale seafloor structure metrics within the MPA network were close approximations of the regional availability of these features for the majority of habitat categories (Fig. 5). The deviation in coverage of each of the categories varied from 0.5% to 119% and the majority of the habitat classes were well represented across the MPAs. The rarer habitat classes, however, were not well represented across the MPAs and their deviations from the regional availability fell outside the 20% threshold chosen (Fig. 5).

### Does the kelp forest monitoring program adequately capture the variability in seafloor structure inside the MPAs and does the monitoring program sample the same seafloor structure inside and outside MPAs?

The cluster analysis of the habitat within the surveyed and our randomly generated transects produced six distinct reef structure classes that were defined by distinctive combinations of geomorphological characteristics. Each covered a certain percentage of the region: low complexity (8%), low to moderate complexity (5%), moderate complexity (26%), moderate to high complexity (11%), high complexity (9%), high complexity on slope (42%). These reef structure classes were significantly clustered at close distances. To reduce the number of unique classes to a number of categories more amenable to the sample size of kelp monitoring transects, a Bray-Curtis dissimilarity distance of 1000 was used. This cutoff value allowed for the incorporation of slightly outlying clusters to be merged into larger clusters without forcing dissimilar clusters to become combined.

**Fig 6:**
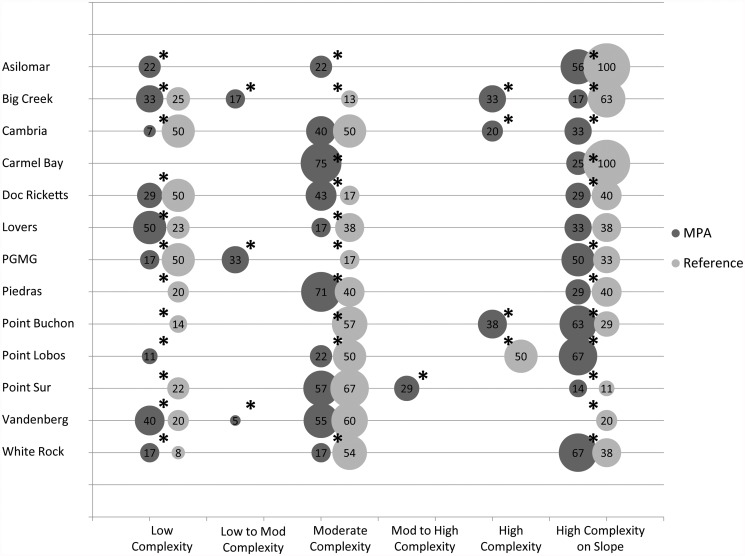
Comparison of the total percentage of each of the habitat classes derived from the cluster analysis within each of the 13 MPAs used in this analysis (light gray) and the percentage of PISCO transects that fell in those habitat classes (dark gray). The size of the circles represent the percentage of transects in each of the corresponding habitat classes and are labeled with the percentage value. The black asterisks above the pairs of circles represent those habitat classes that were not well-represented in the monitoring transects within each of the MPAs. The black asterisks represent those habitat classes that were not well-represented by the monitoring transects based on the 20% deviance threshold.

Using these reef structure classes, we analyzed the percentage of each class found throughout the Central Coast MPAs and compared those percentages to the percentage of baseline monitoring transects that fell in those classes to determine how well the surveyed transects sampled the rocky reefs representatively within the MPAs. In most MPAs, the monitoring sites did not sample the reef structure classes proportionately (Fig. 6). In some MPAs some habitat classes were poorly represented. For example, in the Carmel Bay SMCA, the “moderate complexity” class was over sampled while the “high complexity on slope” class was under-sampled by large percentages (46% and 43%, respectively). In other MPAs, reef structure classes were not sampled at all. In the Point Lobos SMR, the second most predominant class was not sampled and the third most predominant class in the Big Creek SMR was not sampled.

**Fig 7:**
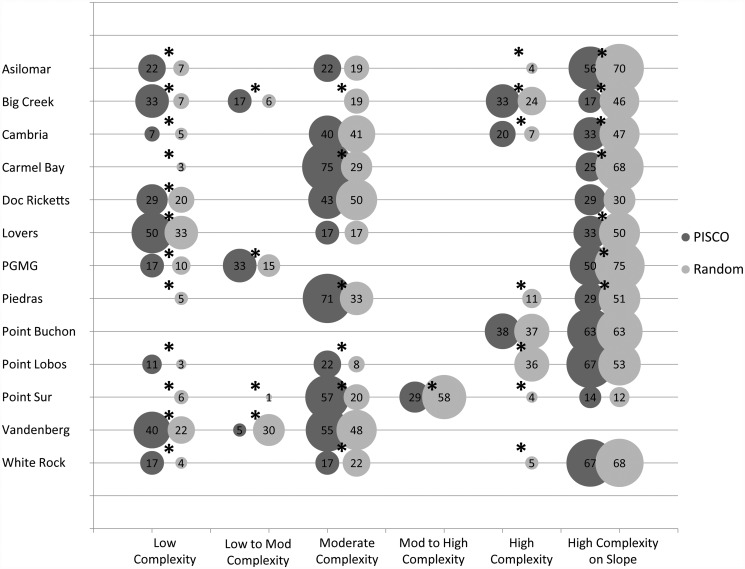
Comparison of the habitat represented in the MPA monitoring transects (dark gray) and the habitat represented in the reference site transects (light gray) for each of the 13 MPAs looked at for this analysis. The size of the circles represent the percentage of transects in each of the corresponding habitat classes and are labeled with that percentage. The black asterisks above the pairs of circles represent those habitat classes that were not proportionately sampled within each MPA and their corresponding reference sites *Note: transects represented in this figure are only those containing fish data. The invertebrate and algae transects were excluded from this analysis.

Using the same reef structure classes from the cluster analysis that were used to evaluate the representation of MPA habitat in the PISCO monitoring design, we compared representation between the MPA sites and their corresponding reference sites. Habitat classes were sampled disproportionately between MPAs and reference sites in all thirteen MPAs (Fig. 7). For example, three classes of reef structure were sampled within the Asilomar MPA but only one of those classes was captured in the reference site. Overall, the representation of reef structure transects in the reference areas was fairly disproportionate except in a few cases.

## Discussion

Results of this study provide an assessment of how well habitat categories targeted for protection by California’s MLPA are represented and replicated across the network of MPAs along the central coast of California. During the designation of these MPAs, there were limited data on the availability (i.e. amount and distribution) of habitats across the region. Since the completion of the California State Mapping Program (CSMP), an unprecedented dataset is now available that allows for the detailed delineation of the habitat categories used in the design of the network, as well as fine-scale geomorphic features. This assessment of habitat representation is a first step in evaluating how well the currently designated network of MPAs representatively include the habitat categories used to achieve representation of ecosystems and their associated biodiversity in the Central Coast Region of the statewide network.

At the spatial scale of the Central Coast network of MPAs, our assessment of how well the current network of MPAs representatively (i.e. proportionately) captures the habitat types designated for protection by the MLPA design process indicates mixed results depending on depth. Therefore, we rejected the hypothesis of representation of some habitat categories and not others. For the shallower MLPA depth classes (0–30m and 30–100m), our results indicate that the distribution and abundance of the designated MLPA habitat categories were successfully represented in the network of MPAs in proportion to their availability in the region. On the other hand, at depths below 100m there were significant discrepancies (> 20%) between the MLPA-predicted and the CSMP-derived estimates of both rocky reef and sediment habitat representation. It is likely that proxies of habitat type such as the availability of relatively well-mapped kelp forests and local fishermen knowledge provided a good basis for the distribution of habitat categories (i.e. rocky versus soft bottom by large depth zones) in the shallow nearshore, but less so in deeper waters. One reason for the bigger discrepancies between MLPA-predicted and CSMP-derived habitat coverage in deeper depth zones could be the coarse scale data used in the MLPA-predicted habitat maps to define the rock and sediment habitats in the deeper depth zones [62]. These coarse scale maps only provide approximations of coverage [63] and limitations of the data used to produce the MLPA-predicted habitat maps was known at the time of designation. Gleason et al. [63] acknowledged that representation of these habitat types within the MPAs should be revisited subsequent to the availability of more accurate map products.

The analysis to determine how well regional geomorphic classes based on finer scale topographic metrics, which were not considered when the MPAs were designed, showed that there was good representation within the Central Coast network of MPAs for the more abundant classes. On the other hand, the fine-scale geomorphic classes that were rarer throughout the region were not sufficiently represented. It is somewhat surprising that the finer-scale categories of reef structure were so well represented in the shallow depths, given the lack of knowledge of these features from traditional sources. One implication of this result is that the finer scale geomorphic classes are sufficiently ubiquitous such that simply capturing hard bottom was sufficient to ensure representation of these higher resolution seafloor structures as well. In addition, variation in the abundance of the fine-scale categories appears to occur at large geographic scales. Therefore, by distributing these large MPAs across the broad geographic variation in coarse-scale habitat categories (i.e. rock and sand at each depth zone) these finer scale geomorphic classes were representatively included in the network.

When the substrate cover in the MLPA-predicted habitat maps was compared to the substrate coverage in the CSMP-derived habitat maps at the scale of individual MPAs there are much larger discrepancies in the representation of habitat, such as the under-representation of rock in the MLPA-predicted substrate maps of the Piedras Blancas MPA (Fig. 4). The under-representation or over-representation of habitat can have very important consequences with respect to both the design and effectiveness of the MPA network. Over-representation of specific habitat classes can have detrimental social or economic impacts. For example, if disproportionately greater amounts of habitat essential to commercially important species are protected within MPAs (e.g., over-representation of rocky habitat, which the majority of commercially important rockfish utilize), the fishery could suffer financial hardship. Under-representation of habitats could also have negative effects if inadequate amounts of critical habitats are not protected. A key goal of the network is to ensure that young produced in one MPA contribute to the larval replenishment of populations from one MPA to the next. This “larval connectivity” is a key element of MPA networks [11, 13, 15, 19, 14, 26, 33]. For populations to contribute to such a network, sufficient habitat to support those populations needs to be included in enough replicate MPAs that contribute to the network. The MLPA design process used cumulative species-area relationships to identify the minimum area of habitat to contribute to a network (where 90% of the species richness of a community is included in the minimum area) [55]. In this design criterion, the replication of four MLPA habitats was specified; 0–30m kelp forest/rocky reef, 0–30m sediment, 30–100m rocky reef, and 30–100m sediment. Our assessment of how well these four habitats are replicated throughout the region shows that, although there were some large discrepancies in the area of habitat within individual MPAs compared to the predicted coverage of those habitats, there is adequate replication of three of the four habitats across the network to meet the design guidelines of the MLPA. The only habitat that is not adequately replicated is sediment habitat in the 30–100m depth range. This result is likely due to the large area (26 km^2^) required of a replicate of this habitat relative to the typical size of MPAs across the network despite the abundance of this habitat in the region.

We considered only finer scale features and geomorphic variation across rocky reefs, not the soft bottom. However, more studies have also identified ecologically important variation in sediment habitats. For example, rippled scour depressions (RSDs) are features that contain coarser grained sediment and are depressed relative to the surrounding sediment [64]. Davis et al. [65] showed that these sediment features are also adequately represented across the Central Coast MLPA region.

Because of the inability of traditional, vessel-based seafloor mapping to collect data in the shallow (0–5m) surf zone in the nearshore environment along the coast, the use of seafloor data to estimate the distribution of habitats may be an under-representation of the rocky reef in the 0–30m depth class. Seafloor mapping is usually terminated at shallower depths due to navigational hazards such as the presence of emergent rocks, thick kelp canopy, or unsafe wave environment. Most of these impediments to surveying are usually indicators of subsurface rock. Therefore, the benthic maps for the state waters often end slightly offshore from the coastline in many areas where there is most likely rocky habitat. The development of new sampling methods and platforms that allow mapping of these shallow nearshore habitats, or analytical tools that allow for accurate extrapolation of adjacent habitat into these zones, is critical because of the abundance and diversity of species in these habitats and the ecosystem functions and services they produce [66]. Unfortunately, a number of kelp forest monitoring transects were conducted in shallow depths that do not overlap with the seafloor habitat data due to the reasons discussed above and, therefore, were not used in our analyses (~ 40%).

Densities of most marine species are typically correlated with seafloor features [46]. Therefore, the spatial design of MPA monitoring programs must capture this variability of structure to accurately estimate the demographic responses (e.g., abundance, size structure, larval production) of populations to the establishment of MPAs [50, 47, 48, 67]. Our assessment of how well the kelp forest monitoring program adequately captured the variability in seafloor structure classes inside the MPAs showed that most of the reef structure classes within the MPAs were sampled but not proportionately. There was not a single MPA where all reef structure classes were representatively sampled. Therefore, we rejected the null hypothesis that the variation in reef structure was proportionately represented in the monitoring design. In addition, the assessment of how well seafloor structure classes within reference sites outside MPAs matched those sampled within the MPAs showed that there were miss-matches in the seafloor structure sampled. There was not a single MPA where the monitoring program sampled the same proportion of habitats in the reference sites as inside the MPA. Therefore, we again rejected the null hypothesis that the seafloor structure was representatively sampled in the reference sites compared to within the MPAs. If species responses to MPAs interact with habitat variation, these differences could confound comparisons of population trajectories over time inside and outside of the MPAs and conclusions regarding species responses to MPA establishment. In fact, taking into account the variation in reef structure greatly alters abundance estimates of fish species compared to methods that assume all rocky reef provides the same quality of habitat. The kelp forest monitoring program in the Central Coast could adjust the locations of transects to ameliorate the effect of habitat differences on estimates of species responses to MPAs. Spatial designs of future monitoring studies should capitalize on the availability of seafloor maps to enhance the statistical power of monitoring studies to detect population responses.

Using metrics derived from seafloor maps to evaluate MPA design has fundamental limitations. This analysis focused on a small subset of variables (geomorphology and depth) that contribute to the characterization of habitat. However, habitat is ultimately defined by the collection of organisms associated with it and seafloor structure is only one set of variables that can influence the distribution and abundance of species across the region. Oceanographic variables, such as currents, water temperature and nutrients that characterize areas of coastal upwelling, riverine input, or differing levels of swell exposure can greatly influence the composition of species that constitute ecosystems. Similarly, biogenic habitat structure such as kelps and corals also contribute to variation in species assemblages that define habitats [53, 51]. Ultimately, how well an individual or network of MPAs representatively includes habitats and biodiversity should consider the greater breadth of oceanographic, geomorphological and biological characteristics that collectively define an ecosystem [53, 51]. As stated previously, the classification of seafloor habitat is an important first step in evaluating the placement of MPAs and can be combined with other measures of habitat quality to create predictive models of species abundance across the region using data generated by oceanographic and ecological monitoring studies.

## Conclusions

Our application of spatially explicit geomorphic metrics derived from seafloor maps proved to be a valuable approach in evaluating the design (habitat representation and replication) of the MPA network along the central coast of California. We found that how well habitat representation and replication was achieved differed by spatial scale and depth. Habitats were best represented and replicated at shallower depths at the scale of the entire network, and were not as well achieved at the scale of individual MPAs and at deeper depths. We also found that the spatial design of ecological monitoring programs (kelp forest surveys) designed to evaluate ecological responses to MPA establishment would have benefited greatly by the existence of high resolution seafloor maps when those surveys were designed. As marine conservation continues to move in the direction of ecosystem-based management and the designation of marine protected areas (MPAs), there is a need for methods that facilitate the design of MPA networks and to evaluate the likelihood of their design in meeting their conservation and management goals [68]. Because of the lack of complete information on the distributions of species and the processes that maintain diversity, populations, species, and ecosystems [69], environmental surrogates that are linked to the maintenance of biodiversity are used in place of complete information [68]. The variables derived from multibeam bathymetry data used in this study (i.e. substrate type, depth, habitat complexity, etc.) have been shown to be important to many marine species and overall biodiversity and, therefore, can be used as a helpful and initial method of evaluating the design of MPA networks and studies of their evaluation. In addition, the generation of species-habitat relationships with the derivatives of the multibeam bathymetry data can be used to further our understanding of how the variation in seafloor structure affects the distribution and population sizes of species for which MPAs are created to protect.

## Supporting Information

S1 File Contains Appendices A and B. (DOCX)
