# Supplementary material for: Assessment of Habitat Representation across a Network of Marine Protected Areas with Implications for the Spatial Design of Monitoring
Source: PLoS One. 2015 Mar 11;10(3):e0116200. doi: 10.1371/journal.pone.0116200 (PMC4356544; doi:10.1371/journal.pone.0116200)
Supplement: S1 File — (DOCX) [file pone.0116200.s001.docx]

Appendix A: Application of digital elevation models (DEMs) to classify MLPA habitat categories.

To distinguish between rock and sediment, vector ruggedness measure (VRM) grids were created from the DEMs using the Terrain Tools toolbox in ArcGIS 9.x [Sappington et al. 2007]. VRM is a measure of terrain ruggedness using vector analysis where the 3-dimensional orientation of the grid cells is taken into account, allowing for variation in slope and aspect [Hobson 1974]. The values associated with VRM vary from 0 (flat, smooth areas) to 1 (areas of higher complexity). Because rock is often more complex than the surrounding sediment, VRM can be used to help distinguish between rock and sediment areas (i.e. "rough" and "smooth" areas, respectively). VRM was, therefore, used as a proxy for "rock" and "sediment."

From the VRM analysis, a binary raster was created with "0" signifying soft sediment and "1" signifying rocky substrate. The breakpoints for these two classes were based on a threshold of VRM that captured the majority of the "rock" without erroneously classifying artifacts or sediment features. Validation of this classification was conducted as follows: 100 random points were placed throughout each survey block and the substrate underlying those points was visually classified within ArcGIS as either "sediment" or "rock." The visual classification was then compared to the results from the VRM classification to determine the accuracy of the habitat classification. Accuracies greater than 90% were considered sufficient. If the accuracy fell below 90%, the site was reclassified by adjusting the threshold value of the VRM. When necessary, a substrate mask was used to mask out any problematic areas.

# References

Hobson ES (1974) Feeding relationships of Teleostean fishes on coral reefs in Kona, Hawaii. Fish. Bull. 72: 915-1031.

Sappington JM, Longshore KM, Thomson DB (2007) Quantifying landscape ruggedness for animal habitat analysis: a case study using bighorn sheep in the Mojave Desert. J. Wildl. Manage. 71: 1419-1426.

Appendix B: Application of DEMs to classify the seafloor into fine-scale reef categories.

Slope was calculated using the Spatial Analyst extension in ArcGIS 9.x [ESRI 2012]. Slope is calculated by determining the max slope value between an individual cell in a DEM and its eight neighbors. These slope values were then classified into the slope categories from the deep water marine benthic classification scheme: "Flat", "Sloping", "Steeply Sloping", and "Vertical" [Greene et al. 1999].

Rugosity was calculated using the rugosity calculator within the Benthic Terrain Modeler toolbox [Wright et al. 2012] in ArcGIS 10.x [ESRI 2012]. However, rather than using one threshold value to distinguish between rock and sediment, multiple thresholds were chosen to distinguish between differing degrees of "ruggedness" (i.e. very low, low, moderate, high, very high) [Greene et al. 1999].

Topographic position index (TPI) is a measure of relative elevation, which indicates the position of a given point in the overall surrounding landscape. TPI can be used to identify and delineate landforms such as peaks, ridges, cliffs, slopes, flat plains, and valleys, and is calculated by comparing the elevation of each cell in the DEM to that of its neighborhood. Because the neighborhood size can be adjusted, TPI can be calculated at various scales. For this analysis, we calculated TPI at two neighborhood sizes using the bathymetric position index (BPI) tool within the Benthic Terrain Modeler toolbar (BTM). We calculated TPI at 20m (fine scale) and 50m (broad scale) to look at features on these two scales, which have been shown to be good predictors of fish distribution [Young et al. 2010]. These TPI grids were then standardized and classified into 6 "slope position" landscape feature values based on the relative elevation and slope of the cells following the classifications of Iampietro et al. [Iampietro et al. 2005]: "Valley/Crevice", "Lower Slope", "Flat/Plain", "Middle Slope", "Upper Slope", and "Peak/Ridge".

# References

ESRI (Environmental Systems Resource Institute) (2012) ArcMap 9.3. ESRI, Redlands, California.

Greene, HG, Yoklavich MM, Starr RM, O'Connell VM, Wakefield WW, et al. (1999) A classification scheme for deep seafloor habitats. Oceanol Acta 22(6): 663-678.

Wright DJ, Pendleton M, Boulware J, Walbridge S, Gerlt B, et al. (2012) ArcGIS Benthic Terrain Modeler (BTM), v.3.0, Environmental Systems Research Institute, NOAA Coastal Services Center, Massachusetts Office of Coastal Zone Management. Available: <http://esriurl.com/5754>

Young MA, Iampietro PJ, Kvitek RG, Garza CD (2010) Multivariate bathymetry-derived generalized linear model accurately predicts rockfish distribution on Cordell Bank, California, USA. Mar Ecol Prog Ser 415: 247-261.

Iampietro, PJ, Kvitek RG, Morris E (2005) Recent advances in automated genus-specific marine habitat mapping enabled by high-resolution multibeam bathymetry. Mar Technol Soc Ser 39: 83-93.
